# Monotonicity, frustration, and ordered response: an analysis of the energy landscape of perturbed large-scale biological networks

**DOI:** 10.1186/1752-0509-4-83

**Published:** 2010-06-10

**Authors:** Giovanni Iacono, Claudio Altafini

**Affiliations:** 1 SISSA-ISAS, International School for Advanced Studies, via Beirut 2-4, 34014 Trieste, Italy

## Abstract

**Background:**

For large-scale biological networks represented as signed graphs, the index of frustration measures how far a network is from a monotone system, i.e., how incoherently the system responds to perturbations.

**Results:**

In this paper we find that the frustration is systematically lower in transcriptional networks (modeled at functional level) than in signaling and metabolic networks (modeled at stoichiometric level). A possible interpretation of this result is in terms of energetic cost of an interaction: an erroneous or contradictory transcriptional action costs much more than a signaling/metabolic error, and therefore must be avoided as much as possible. Averaging over all possible perturbations, however, we also find that unlike for transcriptional networks, in the signaling/metabolic networks the probability of finding the system in its least frustrated configuration tends to be high also in correspondence of a moderate energetic regime, meaning that, in spite of the higher frustration, these networks can achieve a globally ordered response to perturbations even for moderate values of the strength of the interactions. Furthermore, an analysis of the energy landscape shows that signaling and metabolic networks lack energetic barriers around their global optima, a property also favouring global order.

**Conclusion:**

In conclusion, transcriptional and signaling/metabolic networks appear to have systematic differences in both the index of frustration and the transition to global order. These differences are interpretable in terms of the different functions of the various classes of networks.

## Background

**Figure 1:**
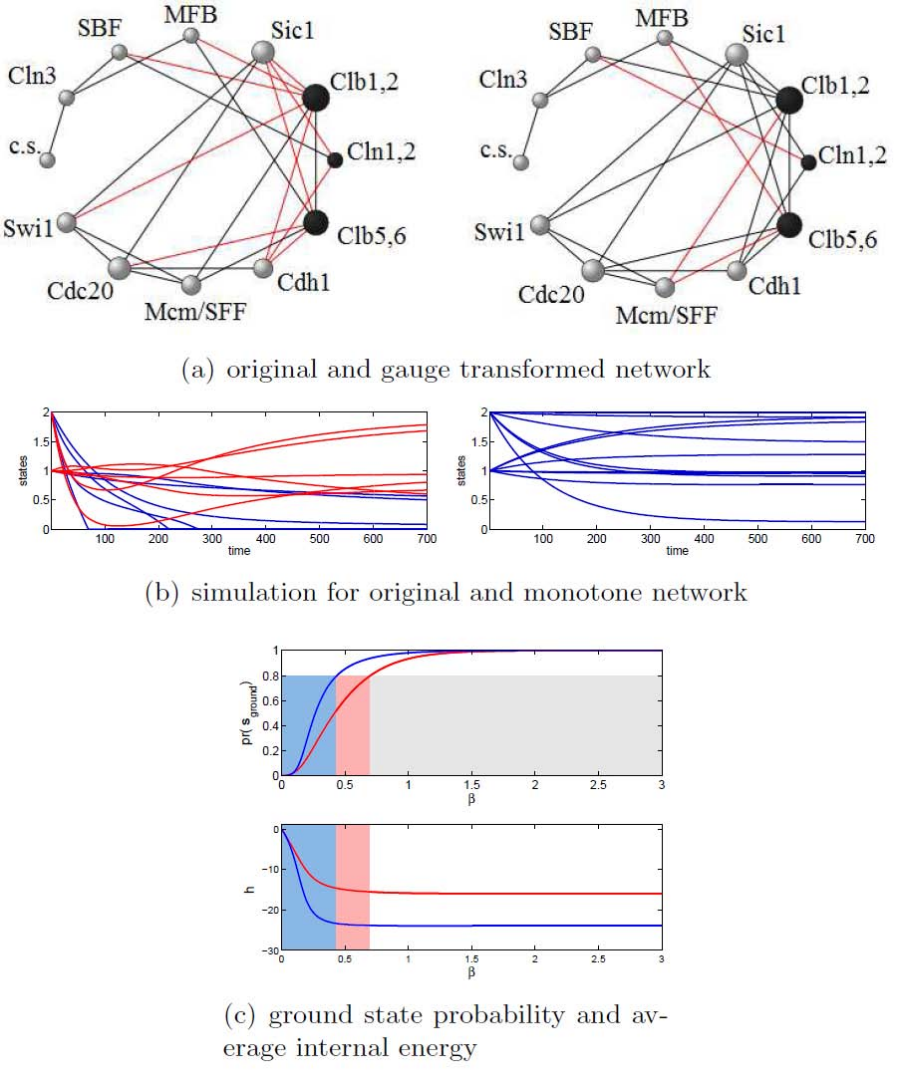
**Yeast cell cycle signed network of **[19]. The undirected graph shown is a symmetrization of the one in [19], in which we also dropped the self-loops. In (a) the application of a gauge transformation to the three nodes in black reduces the number of negative edges (in red) and **s*_σ_***= **1 **becomes a ground state. In (b) a typical response to a perturbation is shown for the yeast cell cycle network and for a monotone network on the same graph: in the second system the order is always maintained in the response (blue trajectories are monotone states). In (c) the probability of being in the ground state p(**s**_*ground*_) (upper plot) and the internal energy (h) (lower plot) are shown for the yeast cell cycle network (red) and for the monotone network (blue) as a function of *β*. As the dimension of the system is small, no mean field approximation is necessary in these calculation. The monotone network achieves order (here p(**s**_*ground*_) > 0.8) earlier with growing *β *and the energy minimum reached is lower. The color bands are meant to highlight the values of *β *for which this happens

For complex systems such as biological networks, rather than a precise description of the dynamics, which requires a quantity of kinetic details rarely accessible in large scale systems, it is often more reasonable to use a minimal representation, such as a graph of interactions between the molecular variables of interest [1-4] and perhaps a sign describing the mode of the pairwise interaction. Such graphical approaches have been extensively used in recent years to model transcriptional [5,6] and signaling networks [7-10]. Apart from biological systems, signed adjacency graphs have been investigated in several different contexts, such as economics [11,12], social balance [13], and in the theory of frustrated spin systems [14,15], see [16] for a survey. In spite of the minimal amount of information it contains, a signed graph can already be used to study dynamical systems properties. Among the various approaches that have been used for this scope, we recall for example the characterizations of multistationarity of [17], stability [18], and the boolean network analysis of e.g. [10,19,20]. In particular, in [21] signed graphs are linked to the theory of monotone dynamical systems [22] and the latter is used as a paradigm to explain the highly predictable and ordered response of biological systems to perturbations. In a biological network, a response to a perturbation propagating incoherently through the network may result in an unpredictable or contradictory behavior of the system, see example in Fig. 1. When its dynamics are always free from such contradictory responses then the system is said monotone [21,22], see Methods for a more rigorous definition. In dynamical systems language, a monotone system exhibits an ordered response because it lacks sustained oscillations and chaotic behavior, thereby rendering the behavior of the system particularly simple. Hence the investigation of how close a biological system is to being monotone has been the subject of intense research in recent years [21,23-26].

From a statistical physics perspective, the problem of determining monotonicity (or near monotonicity) is equivalent to checking when an Ising model with signed interactions has no (or little) frustration [21,23]. In terms of the signed graph, frustration corresponds to undirected cycles having an odd number of negative edges [21]. See also [27] for another recent use of Ising models in the context of complex networks. In this work we are interested in computing the frustration of biological networks of various types: transcriptional, signaling and metabolic. When modeling these different classes of networks as signed graphs, we have to use different levels of resolution: for signaling and metabolic networks we start from a set of stoichiometric reactions and obtain the signed graph by taking the signature of the Jacobian of the corresponding reaction kinetics, hence an edge represents the contribution of a molecular specie to a kinetic reaction, see [8,23,26] and the Methods Section. For transcriptional networks, on the contrary, we model interactions at functional level, i.e., we take an edge to represent the entire action of activation/inhibition of a transcription factor on a target gene, and in doing so we lump together many important molecular steps, from the binding of the transcription factor to the promoter region of a target gene to the final release of the newly synthetized mRNA molecule. Energetically, such complex process is various (or many) times more relevant than a signaling event or a metabolic reaction. Also the corresponding time scales differ by several orders of magnitude [4,28]. Of course, we are forced to use this coarser level of resolution because the stoichiometric details are different for different transcriptional actions, and are not known systematically (see [29] for the only example we know of in this direction). Notice that a similar functional representation, oriented at capturing the "information flow" rather than the "mass flow", is possible also for signaling networks [4,7,9,10]. Although it may elucidate better the causal transfer of "information" along the pathways, it seems less appropriate to describe the energetic content of the biochemical transformations necessary for the propagation of the signal than the stoichiometric level which we use in this paper, see Supplementary Notes in Additional File 1 for a more detailed discussion. In any case, the qualitative difference in the modeling assumptions made should always be kept in mind, and the classes of networks analyzed should be connotated accordingly as "transcriptional, at functional level" and "signaling/metabolic, at stoichiometric level".

Under these assumptions, the frustration index we observe varies considerably according to the type of network analyzed: it is very low for gene regulatory, networks and much higher for signaling and metabolic networks. In this paper we propose an interpretation of this different behavior based on the characteristic "energy" associated to the interactions of a graph. We assume that the costs of the interactions (i.e., the weights of the edges) are all comparable on each class of networks, but not across classes of networks. In particular, transcriptional edges have a much higher cost than the other classes of interactions, and we can speculate that on an evolutionary scale this may have strongly disfavored the development of interactions leading to frustration, i.e., of incoherent or contradictory transcriptional orders. For the "cheaper" signaling and metabolic interactions, instead, such a tight control may not be required, especially since a higher frustration may induce a richer and more complex dynamical behavior.

We know from the theory of Ising models that it is energetically favorable for neighbouring spins to be aligned when the interaction constant is positive and to be antialigned when it is negative. If we associate to the frustration index the global optimum of an "energy" function describing the amount of such unsatisfied interactions, then we can say that networks with low frustration will have a "ground state" (i.e., a global optimum) of lower energy than more frustrated networks. In addition, rather than just focusing on the energy of the optimal configuration, we can average the state of the system over all possible perturbations, and study what is the average frustration of a network. In particular, then, if we take the strength of the interactions of a network as "cooling" parameter, we can use statistical physics arguments [15] to describe how the probability of occupancy of the global minimum of the energy varies with the interaction strength, and therefore how monotonically a network behaves in average in response to random perturbations. What we observe is that the more frustrated signaling/metabolic networks achieve "order" (i.e, tend to populate their global minimum of energy) in a range of interaction energies which is lower than for the transcriptional networks, meaning that these networks (in average) tend to respond to perturbations as coherently as they can even for moderate values of energy. This behavior partially compensates for the higher frustration, which, as already mentioned, might be instrumental to the achievement of more complex dynamics than those required for the transcriptional networks. The transcriptional networks, on the other hand, only contain strong interactions and are therefore not concerned with the lower energetic regime. Coherently, they show a topological structure richer in tree-like subgraphs, which disfavor the transition to ordered behavior, and which are absent in the other classes of networks.

That signaling and metabolic networks may require a lower energetic content to experience a transition to ordered behavior is also confirmed by the structure of their energy landscapes which, unlike for the transcriptional networks, lack high and neat energetic barriers around the global optima, meaning that reconfiguration to the ground state can be easily achieved even at modest energies.

## Results

The representation of a biological network as an n-dimensional signed adjacency matrix is given by a matrix  of elements *J*_*ij *_∈ {±1, 0}, *i*, *j *= 1,..., *n*.  is assumed symmetric (i.e., the frustrated cycles we seek are in the underlying undirected graph), and with zero diagonal (i.e., no self-loops), see [21] and the Methods Section for details on the formulation of the problem. As explained in the Methods, for a stoichiometric system we can assume that  corresponds to the signature of the Jacobian linearization around an equilibrium point. For networks represented as functional activatory/inhibitory actions, the interpretation is even more straightforward. Coherently with our choice of model, we assume that also the perturbations affecting the system around its equilibrium point are of unit magnitude in each component, *s*_*i *_∈ {±1}, *i *= 1,..., *n*. In correspondence of a vector **s** = [*s*_1 _... *s*_*n*_]^*T *^of such signed perturbations, or "spin" variables, let us consider the "energy" function(1)

which expresses the total cost associated to the perturbation **s**. Assuming that all interactions of a network have the same strength, |*J*_*ij*_| = 1 whenever *J*_*ij *_≠ 0, the cost of each interaction depends on the sign of each nonzero *J*_*ij*_: for *J*_*ij *_> 0 (activator) the aligned *s*_*i*_, *s*_*j *_spin configuration is more energetically favorable (-*J*_*ij*_*s*_*i*_*s*_*j *_= -1 < 0) than the antialigned one (-*J*_ij_*s*_*i*_*s*_*j *_= 1 > 0) and viceversa for *J*_*ij *_< 0. Of all 2^*n *^possible spin assignments, those respecting monotonicity will be such that *J*_*ij*_*s*_*i*_*s*_*i *_> 0 on each edge of the graph, i.e., those contributing to minimizing *h*(s). A spin system is said frustrated when not all these constraints *J*_*ij*_*s*_*i*_*s*_*j *_> 0 can be satisfied simultaneously by any assignment. Computing how far a given network is from being monotone corresponds to computing the ground state **s**_ground_, i.e., the spin assignment that globally minimizes (1). It has been shown [23] that this is an NP-hard problem, equivalent to the MAX-CUT problem or, in terms of the Ising model, to computing the exact frustration index of the network [21,30], call it *δ*. In [26] (see also Supplementary Notes in Additional File 1 for a quick recap), we proposed efficient heuristic algorithms providing fairly tight upper and lower bounds for *δ *in biological networks of the size of the thousands nodes. From the theory of monotone systems (see [21,22] and the Methods),  is monotone if and only if there exists a diagonal signature matrix *D*_*σ *_(i.e., a matrix having on the diagonal the vector *σ *of elements *σ*_*i *_∈ {±1}) such that  has all nonnegative entries, see Lemma 2.1 in [22]. _*σ *_and  have different sign patterns but the same frustration index *δ*, as *D*_*σ *_is a change of sign through a cut set of the graph of  and such "gauge transformations" *D*_*σ *_[31] leave the sign of each cycle of the graph (and hence *δ*) unaltered.

Let us consider first as an illustrative example the yeast cell cycle network introduced in [19] in the context of boolean networks, see Fig. 1. With respect to the original graph of [19], we drop the self-loops and consider the underlying undirected graph (only a pair of edges is incompatible with this symmetrization of the adjacency matrix). The number of negative signs on the symmetrized adjacency matrix  is 10. However, a gauge transformation on the three nodes Cib1,2 Clb5,6 and Cln1,2 yields a _*σ *_with only 4 negative edges, which is a global optimum for the frustration index *δ*, see Fig. 1(a) . The presence of frustrated cycles in a network leads to a lack of coherence in the response of the system to perturbations.

This can be observed in Fig. 1(b), where the response of the yeast cell cycle and of a monotone network built on the same graph are compared. The behavior of the non-monotone cell cycle network is less predictable and potentially contradictory (see also Fig. S2 for analogous considerations on the simpler feedforward loop example [5]). It is then important to have an estimate of how close a true network is to being monotone i.e., frustration-free. Our algorithms allow to obtain a _*σ *_with as low as possible number of negative signs also for large-scale networks. This number is typically close to *δ*, meaning that it is now much easier to localize on the graph of _*σ *_the potentially frustrated edges (or, more properly, the frustrated cycles). Another consequence is that the candidate ground state for _*σ *_that globally minimizes (1) is now straightforward to identify, as it corresponds to the "all spins up" configuration, call it **1**. Hence, the candidate ground state for the original  can be found reversing the gauge transformation: **s**_*ground *_= *D*_*σ *_**1**. Approximate values for the frustration index *δ *and for the corresponding energy minimum  not very far from the true ones can therefore be computed.

### Frustration in large-scale biological networks

**Figure 2:**
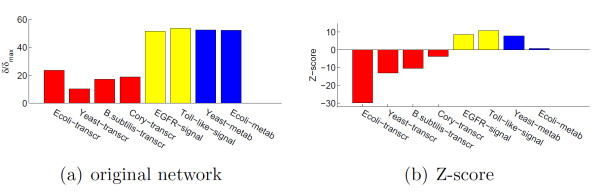
**Frustration index of the 8 biological networks listed in Table 1**. In (a) the ratio δ/δ_max _is based only on the number of nodes and edges of a network and shows that the frustration index is much lower for transcriptional than for signaling/metabolic networks, see Table 2. The Z-score in (b) takes into account also the topology of a network. Again, the transcriptional networks are more monotone (i.e., less frustrated) than expected from a null model, while metabolic and in particular signaling are less monotone (i.e., more frustrated) than expected. P-values for the Z-score are in Table 2.

**Figure 3:**
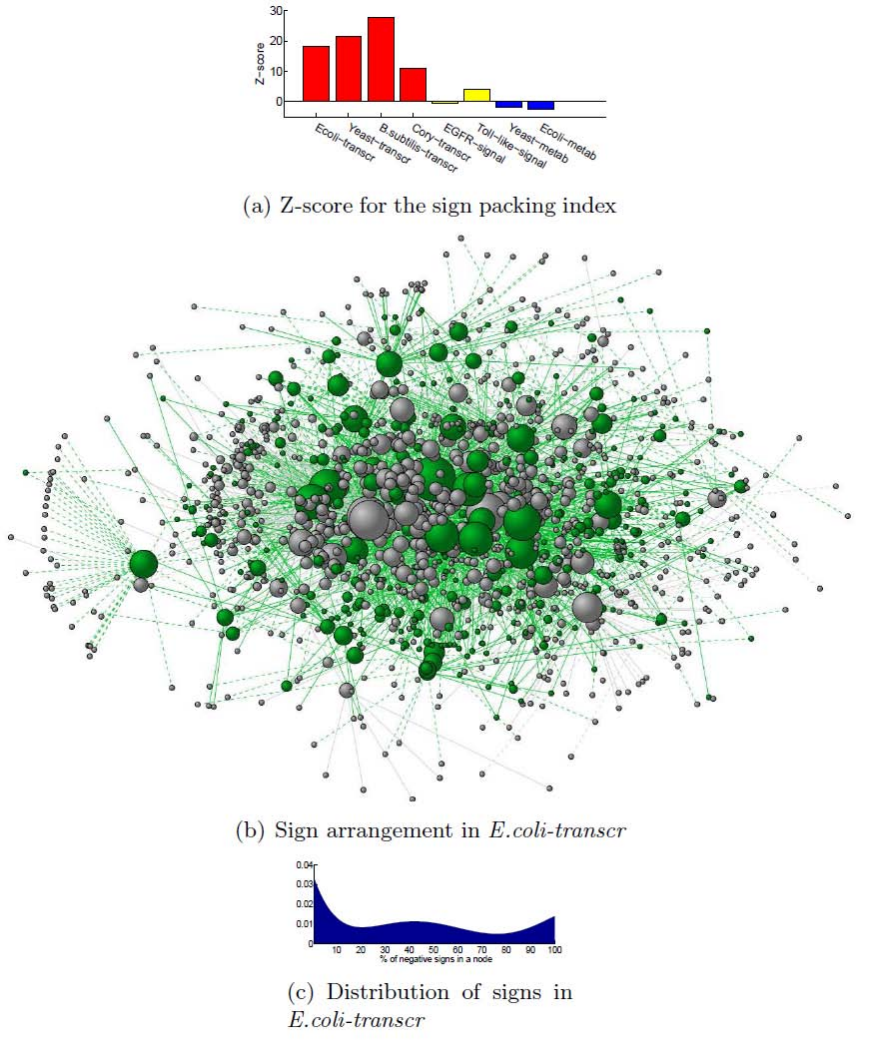
**(a) Z-score for the sign packing index (see Supplementary Notes in Additional File **1** for a definition)**. The 4 transcriptional networks have sign arrangements on the nodes that are significantly asymmetric, hence improving their frustration index. (b) Representation of the sign packing property on the *E.coli * transcriptional network. The nodes significantly enriched in either positive or negative edges are shown in green (the size is proportional to their connectivity). The distribution of negative edges (dashed) is shown in (c). This graph should be compared with the random sign assignment of Fig. S6.

For the eight large-scale biological networks listed in Table 1, four transcriptional *(E.coli, Yeast, B.subtilis *and *Corynebacterium*), two signaling *(EGFR *and *Toll-like*) and two metabolic *(E.coli *and *Yeast *networks), we considered the corresponding signed graphs (see Tables S1-S2 for further details on the networks and the Supplementary Notes in Additional File 1 for the construction procedure followed) and estimated δ through the algorithms mentioned above, see Table 2. The theory of signed graphs provides us with a theoretical upper bound on the frustration index (see Supplementary Notes in Additional File 1), call it *δ*_*max*_, which is a function only of the number of nodes, edges and cycles of the networks. The ratio *δ/δ*_*max*_, Fig. 2(a), shows a marked difference between transcriptional networks and signaling/metabolic networks, with the former exhibiting a consistently lower level of frustration than the latter. The upper bound *δ*_*max*_, however, disregards completely the topological structure and the sign arrangements of a network. To take into account also these parameters, we constructed a null-model of the networks, obtained by randomly reshuffling the signs of the edges, while maintaining the same number of positive and negative edges of the original graph, see Supplementary Notes in Additional File 1 for more details. For the Z-score of this null model, a negative value means that the edges are arranged in order to decrease frustration. We can observe in Fig. 2(b) that all the transcriptional networks have a negative Z-score, and only them (p-values of the Z-score in Table 2). The characteristic property of the transcriptional networks that enhances monotonicity is the tendency of many nodes to have a skewed distribution of signs in their edges, see Fig. 3. Up to a gauge transformation, in fact, highly asymmetric sign distributions correspond to highly positive sign concentrations, hence closer to monotone than random sign distributions. The "packing" of signs on certain nodes is primarily due to the mode of action of the transcription factors. Although dual role (i.e., both activator and repressor) transcription factors exist in both prokaryotes and eukaryotes [32,33], most transcription factors seem to be playing only one role on their target genes. The nature of this single role is sometimes associated to the regulatory domains found on the proteins, especially for activator domains, which are usually enriched in proline, glutamine or acidic amino acid residues [34-36]. The dual role transcription factors are usually able to perform opposite functions according to possible different positions of their binding sequence with respect to the gene sequence, or according to different cellular contexts, or simply enhancing only the formation of the closed complex DNA-RNA polymerase [32]. For example, 71% of the *E.coli *transcription factors function only as activators or repressors, Fig. 3(b). The ontological analysis of the dual role transcription factors is significantly enriched for categories such as interfacing the cell with its extracellular environment and for the elaboration of external stimuli (see Table S5). Hence mixed role transcription factors are more often mediating signaling events than their single role counterparts. It is shown in Fig. 3(a) that all transcriptional networks (and only them) have sign arrangements on the edges that are more skewed than expected (with respect to a binomial distribution model, see Supplementary Notes in Additional File 1 and Table S6) and also this property contributes to their monotonicity (Fig. S5). Another structural difference between transcriptional and signaling/metabolic networks is the overrepresentation in these last classes of short frustrated cycles. As explained in the Supplementary Notes in Additional File 1, this characteristic is encoded in the level of detail (stoichiometric) that we choose to represent our networks, and expresses the lack of global monotonicity of a biochemical reaction involving multiple reagents, see also [21,23,24,26].

**Table 1:** Networks used in this study.

Network			*n*	*m*	leaves	description
transcriptional						level of detail: functional

*E.coli*			1475	3320	556	gene regulatory network of the E.coli, from RegulonDB database, ([42], http://regulondb.ccg.unam.mx), version 6.3.
*Yeast*			690	1082	348	gene regulatory network of S.cerevisiae, from [5]
*B.subtilis*			918	1324	528	gene regulatory network for Bacillus Subtilis, assembled by [43]
*Cory*			344	366	264	Corynebacteria gene regulatory network (experimental interactions only). Assembled by [44]

signaling						level of detail: stoichiometric

*EGRF*			330	852	12	Epidermal Growth Factor Receptor pathway. Created by [45]
*Toll-like*			679	2204	59	Signaling network for the Toll-like-receptor. Assembled by [46]

metabolic						level of detail: stoichiometric

*E.coli*			757	6116	84	metabolic network of E.coli, from [47]
*Yeast*			797	4436	17	metabolic network of the yeast S.cerevisiae. Assembled from [48]

*n *and *m *are the number of nodes and edges of the directed graph; "leaves" is the number of nodes not involved in any undirected cycle. More details on the networks are provided in Tables S1-S2.

**Table 2:** Data for the frustration index *δ*.

Network	**δ**_***low***_	**δ**_***up***_	**δ**_***max***_	**δ**_***null***_	**σ**_***null***_	**Z**_***score***_	Pvalue
transcriptional							

*E. Coli*	365	371	1579	662,86	9,77	29,86	*p*≪ 10^-100^
*Yeast*	41	41	401	116,67	5,83	12,98	*p *= 8 · 10^-39^
*B. Subtilis*	71	71	415	139,73	6,53	10,52	*p *= 3,5 · 10^-26^
*Cory*	9	9	48	71,15	2,16	3,76	*p *= 8,3 · 10^-5^

signaling							

*EGFR*	183	193	375	149,75	5,01	-8,62	*p *= 3,3 · 10^-18^
*Toll-like*	401	468	873	384,92	7,70	-10,78	*p *= 2,1 · 10^-27^

metabolic							

*Yeast metab*	670	747	1421	667,42	10,3	-7,72	*p *= 5,6 · 10^-15^
Ecoli metab	912	1017	1944	1006,9	12,73	-0,79	*p *= 0,21

*δ*_low _and *δ*_up _are the computational lower and upper bounds found for *δ *by the algorithms described in Supplementary Notes in Additional File 1; δ_max _is the theoretical upper bound. The Z-score statistics for the frustration index δ is based on the null model obtained reshuffling the signs (see Supplementary Notes in Additional File 1). δ_null _and δ_null _are mean and standard deviation of the null model. The Z-score compares this statistics with the "true" δ (here we use δ_up_, more conservative).

### Average frustration and ordered response

The values of δ and *h*(**s**_*ground*_) alone are not enough to characterize how monotonically the system behaves *in average*. In fact, the energy landscape of frustrated Ising spin systems is known to be usually rugged [37,38], and the presence of a single deep minimum in (1) is not enough to guarantee that the energy averaged over all configurations **s **(corresponding to all possible multinode perturbations) is indeed more negative than in other systems whose energy landscape is characterized by valleys which are maybe less deep but with larger basins. In other words, to characterize how monotone is the response of the system to arbitrarily complex perturbations we have to consider the average value that *h*(**s**) assumes over all possible spin assignments, weighted by the probability of each **s**. This "internal energy", call it ⟨*h*⟩, is an indicator of how coherently the system is behaving in average: the more negative ⟨*h*⟩ is, the less the responses of the system to perturbations are "contradictory" at some fan-in node or along directed cycles. Denote with  the partition function of the system, *β *∈ ℝ_+_. As usual in statistical physics, the partition function *Z *is the normalization factor that renders the frequencies of the various spin states true probability densities. For spin systems, *β *has the meaning of an inverse temperature and it is normally used as "cooling" parameter, i.e., when *β*→ ∞ the probability of the state **s**, *p*(**s**), tends to concentrate on the ground states: *p*(**s**_*ground*_) → 1 as *β *→ ∞. In the context of biological networks, the temperature is taken as ~ 298 K and it is not a varying parameter. However, we can use *β *to describe the strength of the interactions of a network. Recall that in forming the energy (1),  was taken as a signed adjacency matrix with all interactions equal to 1, regardless of the nature of the network studied. As a matter of fact, metabolic, signaling and transcriptional interactions are characterized by widely different energetic costs. In particular, if in our stoichiometric representation a metabolic reaction or a signaling event might have a comparable energetic content, a link in a gene regulatory network describes the entire cascade of events in which the transcription of a gene can be broken down and overall its cost is much higher than in the other networks. Hence, in our fixed temperature context, taking into account the interaction cost *β *rescales *h*(**s**) to the "absolute" energy *βh*(**s**). The probability of a given configuration **s**, *p*(**s**) = e^-*βh*(s)^*Z*(*β*)^-1^, is a function of *β *and is maximized in the (usually degenerate) ground state **s**_*ground*_. As for spin systems, , i.e., when *β *is large enough, in average the system will always be found in the configuration **s**_*ground *_which minimizes the energy (1) and which exhibits the least frustration for the network.

**Figure 4:**
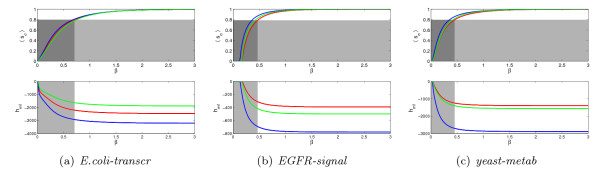
**Computation of the mean field "magnetization" ⟨**s**_σ_⟩ (in the gauge transformed basis) and energy **h**_*mf *_for a transcriptional (*E.coli-transcr*, left panel), a signaling (*EGFR-signal*, middle), and a metabolic (*Yeast-metab*, right) network as a function of *β *(interaction strength)**. The values for the three true networks are depicted in red. In blue and green the same ⟨**s**_σ_⟩ and h_*mf *_for two alternative networks built on the same graph: the exactly monotone network (i.e., with all *J*_*ij *_> 0), and a network with random sign assignments. The gray shaded areas in the upper plot delimit the region ⟨**s**_σ_⟩ ≤ 0.8 i.e., the region in which the response of the system to a generic perturbation results in a low-medium ⟨**s**_σ_⟩. ⟨**s**_σ_⟩ ≥ 0.8 means that in the gauge transformed basis the state **s_σ_**"concentrates" sufficiently well at the ground state, and, correspondingly, the energy is in average sufficiently close to the minimum (lower plots). For the *E.coli-transc *network the threshold ⟨**s**_σ_⟩ ≥ 0.8 is achieved in correspondence of *β *= 0.71, higher than the *β *= 0.46, 0.45 of *EGFR-signal* and *Yeast-metab*. Similar differences are observed in the other networks, see Table S4 and Fig. S7, and are also confirmed in Metropolis-Montecarlo simulations, see Fig. S9

Using *β *as a Lagrange multiplier, the internal energy ⟨*h*⟩ is defined as the expectation value of *h*(**s**),

and expresses this simultaneous weighting of the configurations by their degeneracy and energetic content. The more negative ⟨*h*⟩ is, the more we expect the system to respond coherently to a generic perturbation. For any *β *> 0, ⟨*h*⟩ < 0 and, as *β *increases, ⟨*h*⟩ reaches a stationary value, see Fig. 1(c). For spin systems, small values of *β *represent a regimen where thermal fluctuations are dominant and all states tend to be equally populated. As *β *increases, a spin system usually undergoes a phase transition characterized by the appearance of long range correlations in the expectation values assumed by the *s*_*i*_. For our biological networks, when *β *(i.e., the energetic content of an edge of the network) is too small, the behavior of the network tends to be random (and all states **s** equiprobable) regardless of the monotonicity of the network, a clear obstacle to carrying out any meaningful task. On the other hand, when *β *→ ∞, the probability concentrates exclusively on the ground states (Z(*β*) becomes a Dirac delta function) and the behavior of the system becomes as ordered as its frustration index allows, i.e., the system response is as coherent and coordinated as possible, regardless of the type of perturbation, see Fig. 1(c) . It is then important to see how the probabilities of the various states p(**s**) and the internal energy (⟨*h*⟩) vary as a function of *β *on the various categories of networks under exam. Computing p(**s**) and ⟨*h*⟩ exactly is impossible for systems larger than a few tens of nodes. For larger networks we shall make use of a mean field approximation for heterogeneous networks [39,40]. This approximation, see the "Methods" Section for details, allows to estimate the expectation value ⟨**s**_σ_⟩ in the gauge transformed basis, and the corresponding mean field energy *h*_*mf*_. Fig. 4 shows the behavior of ⟨**s**_σ_⟩ and *h*_*mf *_for a transcriptional, a signaling and a metabolic network as function of *β*. In all three cases, the population concentrates in the ground state when *β *grows, and, correspondingly, *h*_*mf *_achieves its global minimum. The true characteristic value of the interaction strength *β *at which each of the classes of networks should be computed is unknown, except for the suggestion that *β*_*transcr *_≫ *β*_*signal *_~ *β*_*metab*_. Interestingly, as *β *grows, the transcriptional network is slower to reach its energetic minimum than the other two networks, and likewise for the other 5 networks, see Table S4 and Fig. S7. This shift of the coherence barrier towards the low energetic regions is a consequence of the topology of the networks. In fact, as can be seen on Fig. 4, also the completely monotone networks built on the same graphs (blue curves) as well as other networks with random sign assignment to the same edges as our  (green curves) present the same characteristic patterns in spite of different δ. A feature behind this difference is the already mentioned overrepresentation of closed undirected cycles of short length in the structure of metabolic and signaling networks. Also the lower dispersion in the number of connectivity degree classes *k *in these networks contributes to the quick convergence of ⟨**s**_σ_⟩ to 1. However, the main reason behind the different thresholds for *β *is the presence or less of leaves in the graph. For example, the *E.coli *transcriptional network has 38% of the nodes that are not involved in any (undirected) cycle, see Table 1. Dropping these nodes and concentrating on the 2-core of the undirected graph, we obtain mean field plots in which the threshold for order is lower, and similar to those of the signaling/metabolic networks, see Fig. S8. All of our transcriptional networks have a high percentage of nodes that are leaves, much higher that the signaling/metabolic networks, see Table 1. The complete lack of feedback, characteristic of tree-like subnetworks, disfavours the achievement of a globally ordered behavior, which is more easily achieved when short cycles, like the 3-node motifs of signaling/metabolic networks, are abundant. This is expected from the theory of spin systems, where long-range correlations are more easily obtained in dense graphs than in sparse ones. Of course adding leaves to a graph does not change its monotonicity properties (a tree is always monotone).

The qualitative difference in the phase transition to order between transcriptional and signaling/metabolic networks suggests an interpretation coherent with the different energetic content associated to the classes of networks. In fact, we can say that since *β*_*transcr *_is high, it is much less plausible for a transcriptional network to be operating in a regimen of low *β *than it is for signaling/metabolic networks. On the contrary, for these last two classes of networks, it is not unlikely to have interactions of medium-low strength. Hence it gets much more important that ⟨**s**_σ_⟩ → 1 even in correspondence of moderate values of *β*, because this helps in maintaining a coherent behavior in response to perturbations, as required in order to carry out correctly a biological task.

### Sampling the energy landscape

**Figure 5:**
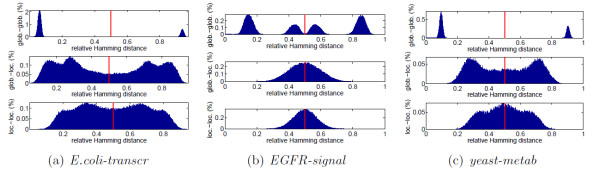
**Relative Hamming distance (number of spin flips over the number of nodes) between pairs of minima found by the algorithms for a transcriptional, *E.coli-transcr*, (a), a signaling, *EGFR-signal*, (b), and a metabolic, *Yeast-metab*, (c), network**. The top plots refer to pairs of global minima; the middle row to pairs global-local minima and the bottom row to pairs of local minima. In all three networks the red line delimits the global symmetry axis of the spin assignment (the locations of the minima have a global spin flip symmetry; the different height of the peaks means that an area has been explored less by the random searches of the algorithm, not that they have different "probabilities"). While for *E.coli-transcr* and *Yeast-metab* the minima are concentrated in a single well, which is quite tight and located near the right margin of the histograms (i.e., short interminimum distances), in *EGFR-signal* there are two such wells and they are disjoint and quite broad. In all 3 networks, adding the local minima, the landscape of minima becomes diffuse, with many different local minima located at varying distances from the global ones.

**Figure 6:**
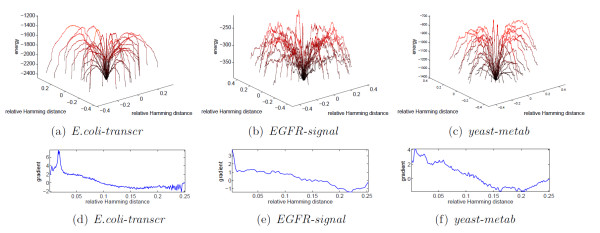
**Montecarlo trajectories connecting a global minimum to its surrounding local minima**. The spin configurations of a global and a local minimum are randomly chosen among those provided by our minimization procedure. The first is mapped in the second by a number of moves (single spin flips) equal to the Hamming distance between the two minima. For visualization purposes, the trajectories are depicted as emanating from a unique point and radially distributed according to a polar coordinate. The vertical axis (and color code) represents the energy, the two remaining axes a relative Hamming distance between spin configurations. The three plots essentially confirm the landscape described in Fig. 5. For *E.coli-transcr*, global and local minima seem to be always separated by a high and steep barrier. In *EGFR-signal *and *Yeast-metab*, the landscape is scattered with different local minima, many of which have energies similar to the global ones, see Fig. S11. This results in some trajectories never emerging from the ordered phase while moving from a minimum to the optimal frustration. The lower row shows the average gradient over 1000 Montecarlo trajectories originating in a global minimum. For *E.coli-transcr *the barriers of the well of global optima is precisely observable in correspondence of the peak of the gradient. For *Yeast-metab *such kinetic traps are less steep. For *EGFR-signal *no clear boundary at all is observable. This, together with Fig. S11, suggests that in the last two networks also spin configurations that are distant from the optimum have cheap routes to converge to the optimal frustration through intermediate low-energy local minima.

Further information, from a different perspective, can be obtained studying the structure of the energy landscape of the different networks [37]. In order to have a picture of how this landscape looks like, we have applied our frustration minimization algorithms to uniformly distributed initial conditions and registered the local and global minima achieved in the process (see Fig. 3 and Table S3). Fig. 5 shows these distributions of minima as a function of the relative Hamming distance. For the transcriptional network of *E.coli *and the *Yeast *metabolic network, the global minima are all localized in a small region, while *EGFR *has two broader valleys of global minima. In all three cases, the global minima are surrounded by many local minima, thus confirming the ruggedness of the landscapes. As can be seen on Fig. S11, unlike *EGFR *and the metabolic network, the local minima of the transcriptional network of *E.coli *tend to have an energetic difference from the global ones which grows linearly with the distance. In addition, the separation between the well of global minima and its surroundings is much more neat in *E.coli *than in the other two networks, as can be seen on the Montecarlo trajectories of Fig. 6 and even more clearly on the average gradient of *h*(**s**) (bottom part of Fig. 6). See also Figs. S10, S12, and S13 for analogous consideration on the remaining 5 networks. Overall, it appears that global and local minima in the transcriptional networks are separated by high and steep energetic barriers, while on the other networks there always exist low-energy routes between random spin configurations and global minima, possibly passing through low-energy local minima. This of course facilitates the achievement of the ground state and the creation of global order even in a regime of moderate values of *β*.

## Discussion

For a gene regulatory network, an edge represents the cost of the entire action of transcription of a gene. This is a complex, multistep process: for prokaryotes, for example, it includes the binding of the transcription factor to the DNA, the recruitment of a polymerase, the unwinding of the DNA helix, the detachment of the σ -factor and the conformational changes in the polymerase preceeding elongation, the release of both the DNA and of the complete mRNA at the termination phase. The energetic cost and time constant of such a complex process are relevant for a cell. Hence, especially in lower organisms, it is natural to expect that in a transcriptional network the genes behave in concert and that the fraction of the gene-gene interactions that contribute to minimizing the energy in response to perturbations is substantially larger than in a metabolic or signaling network, as a frustrated bond costs much more to the cell and its effect lasts much longer. In particular, frustrations manifest themselves on the cycles of the underlying undirected graph of the network as contradictory transcriptional orders. While changing the transcriptional commands is necessary to cope with e.g. different environmental conditions, encoding them as frustrated cycles can easily lead to unpredictable or erroneous dynamical behavior. Therefore, in spite of the presence of certain characteristic motifs leading to frustration (the incoherent feedforward loops mentioned in [5,6] for the *E.coli *and *Yeast *transcriptional networks are common examples), overall the transcriptional networks we analyze are indeed near-monotone. Both the topology and the sign assignments to the nodes of the transcriptional networks contribute to achieve a degree of monotonicity which is higher than expected from null models. On the contrary, incoherent signaling or metabolic actions are energetically much less relevant than a single transcriptional event and can be easily tolerated by the cell, especially since nonmonotone patterns favour a richer dynamical behavior. While the level of detail at which we model our networks (functional for transcriptional networks, stoichiometric for signaling and metabolic networks) certainly contributes to the systematic differences in the frustration index, other factors such as the tendency of the transcriptional networks to have skewed sign distributions are also crucial in attaining a low frustration. It is interesting, then, to notice that in *E.coli *the transcription factors violating this rule are primarily involved in the mediation of external signaling, rather than in regulatory or structural functions (Table S5).

For spin systems, the tendency to satisfy pairwise all interactions grows when the temperature decreases, although in a frustrated Ising spin system all the conditions can never be satisfied simultaneously. In this paper, we consider the strength of the interactions as the key factor that determines the increase in the probability of finding the system in its ground state (i.e., in its least frustrated/maximally monotone configuration). If we parametrize the networks by the interaction strength and study the probability of finding the system response in the ground state as a function of this cost, we observe that for signaling/metabolic networks it is higher than for transcriptional networks in the region of medium/low values of the interactions. This behavior, which is due to the topological structure of the networks and to the energy landscape it determines, could reflect the tendency of signaling/metabolic networks to attain a globally ordered response in spite of the weaker energetic content of their interactions. As such, it helps maintaining coherence of the response in spite of the higher level of frustration of these networks (which, again, favors a richer dynamical behavior). For transcriptional networks, on the other hand, owing to the strong interactions, the regime of low energies is less important, hence tree-like motifs, which hinder the establishment of long-range correlations, are abundant.

A Montecarlo investigation of the energy landscape of the networks [37,41] suggests that transcriptional networks tend to have a more funneled landscape than the other networks (at least around the global optima), with a single deep well of global minima delimited by high barriers, while in signaling and metabolic networks the optima are surrounded by local minima of comparable energy. Order in these classes of networks is favored also by the lack of neat energetic barriers separating local and global optima, which enables the reconfiguration to the global optimum through low-energy paths. 

Several are the *caveat *and limitations of our study. First of all, the different levels of resolution for the different classes of networks may be a source (or *the *source) of the systematic differences we are observing. Hints in this direction come for example from the observation that networks at functional level tend to have less cycles than networks at stoichiometric level (see Supplementary Notes in Additional File 1 for the origin of this fact), and that functional models of signaling pathways may also have asymmetric sign distributions (for example non-specific kinases catalyzing the phosphorylation of various proteins will have many positive edges, while non-specific phosphatases will have multiple negative edges). This is observed to some extent in the functional model of the hippocampal signaling network proposed in [9]. Notice that this network has a large fraction (approximately a third) of interactions representing protein-protein or protein-ligand bindings, to which it is unclear how to associate a sign in an unambiguous manner. The ambiguity of course also propagates to the level of frustration one obtains correspondingly. More generally, we are not aware of any systematic way to map the pathway charts available at stoichiometric level to the functional level, allowing to univocally assign a sign to each edge without at the same time loosing in this process a large part of the molecular species involved. Notice also that the opposite option, namely representing transcriptional networks at stoichiometric level, is *de facto *impossible with our current knowledge.

Another important source of uncertainty comes from the limited coverage of the biological networks currently available. In particular, for the transcriptional networks, the fraction of target genes having at least a transcription factor is below 50% of the genes. Furthermore, our considerations about an higher than expected monotonicity may very well be overturned once more complex organisms (for whom the regulatory mechanisms are expected to be much more complex) are taken into account.

## Conclusion

In conclusion, we have observed that distinct classes of biological networks seem to be characterizable by different features in response to perturbations. At least when we model transcriptional networks at functional level (i.e., as activation/inhibition links) and signaling and metabolic networks at stoichiometric level, we can observe that transcriptional networks appear to be less frustrated than expected and much less frustrated than signaling and metabolic networks, meaning that they might admit highly coherent responses to perturbations. On the other hand, the signaling/metabolic networks seem to have the ability to achieve an average ordered response in a lower range of interaction strengths than the transcriptional networks. We explain the first feature as the need to avoid as much as possible erroneous or contradictory transcriptional actions which would cost much more to the cell than analogous incoherent signaling/metabolic events. The second feature may partially compensate for the higher frustration of these last networks, by lowering the interaction strength needed for a transition to ordered response (in average), and thereby ensuring the effectiveness of this reduced coherent behavior in an energetic range more critical for these classes of networks.

## Methods

**Model formulation: the signed adjacency matrix of a dynamical system **For an n-dimensional system of differential equations(2)

consider the linearization around an operating point x_o_. If **z** = **x** - **x_o_**,

where  is the Jacobian matrix computed at . The system is at rest at , implying that **z **= 0 is an equilibrium point. A perturbation around **x**_o _is then any vector **z **around 0 (assuming both positive and negative values on each of its components *z*_*i*_). For a large-scale biological network it is very difficult to have a precise knowledge of the functional form of *f*(·) or even of the Jacobian matrix . It is often more reasonable to assume that only the sign pattern is known of :

i.e., _*ns *_of elements *J*_*ns*_,_*ij*_ ∈ {±1,0} is the signed adjacency matrix of a directed graph representing our network. Coherently with *J*_*ns*_,_*ij*_ ∈ {±1,0}, also the magnitude of the perturbations **z **is to be considered as unknown except for its sign: **s **= *sign*(**z**), meaning *s*_*i *_∈ {±1} ∀i = 1,... n. The entries *J*_*ns*_,_*ij *_of the matrix _*ns *_represent the effect of the *j*-th variable on the *i*-th variable which can be activatory, *J*_*ns*_,_*ij *_> 0, inhibitory, *J*_*ns*_,_*ij *_< 0, or inexisting, *J*_*ns*_,_*ij *_= 0. In general, this effect can change of sign with the operating point **x**_o _[21], but we shall not consider this scenario here. As a matter of fact, it is worth remarking that for common choices of f(**x**), such as mass-action or Michaelis-Menten, the partial derivatives  have indeed constant sign .

If, rather than in the directed graph of adjacency matrix _*ns*_, we are interested in the underlying undirected graph (resulting by dropping the arrows in the edges), then this is obtained symmetrizing the matrix _*ns *_Denote  such symmetric signed adjacency matrix. The symmetrization operation is always possible as long as edge pairs *J*_*ns*_,_*ij *_and *J*_*ns*_,_*ji *_are compatible, i.e., *J*_*ns*_,_*ij *_*J*_*ns*_,_*ji *_≥ 0. In all of our networks, the symmetrization operation leads to very few or no conflicting signs at all, see Table S1.

### Monotone dynamical system

A partial order in ℝ^*n *^is a signature vector σ = [σ_1 _...σ_*n*_], σ_*i *_∈ {±1}, which defines an order relation among vectors in ℝ^*n*^: *x*' and *x*'' ∈ ℝ^*n *^are said ordered with respect to the partial order . A system is monotone with respect to the partial order *σ *if for any pair of initial conditions *x*'(0) ≤_σ _*x*''(0) one has that *x*'(t) ≤_σ _*x*''(t) for every *t ≥ *0. In terms of the signature adjacency matrix _*ns *_of the Jacobian linearization , a system is monotone if and only if(3)

As explained in detail in [21], the non strict inequality for monotonicity allows to test such conditions (3), rather than in the original directed graph of (2), on its underlying undirected counterpart, in which we conventionally drop the self-loops (for which σ_*i*_σ_*i*_*J*_*ns,ii *_> 0 if and only if *J*_*ns,ii *_> 0, i.e., the order relations (3) are trivial). Therefore, from now on we shall consider only the symmetrized version of _*ns *_,with all diagonal elements fixed to 0, i.e., the matrix . Practically, this symmetrization operation means that we are interested not only to "true" directed cycles and their frustration, but also to multiple directed paths starting and ending on the same nodes (and forming cycles on the underlying undirected graph). See the feedforward examples in Supplementary Notes in Additional File 1 and Fig. S2.

### Mean field approximation in heterogeneous signed networks

Mean field approximations [15] are necessary to compute estimates of quantities such as *Z*, *p*(**s**) and ⟨*h*⟩. The approximation described here is suitable for heterogeneous networks, i.e., networks in which the connectivity of the nodes is not constant. It extends the approach proposed in [39,40] to systems with frustration. For a given signed network , apply first the gauge transformation D_σ _required to minimize the overall number of negative signs on the edges, while maintaining the frustration index *δ *invariant. Denote then *k*(1),..., *k*(£) the £ different connectivity degrees of the nodes of _σ_, of probabilities *p*_*k*_(1),...,*p*_*k*_(£), and ⟨*k*⟩ the average connectivity degree of . The nodes having degree *k *will have a certain distribution of positive and negative edges. Let *k*_*pn*_(1),..., *k*_*pn*_(£) be the differences between positive and negative edges averaged over all nodes of each degree class. As after preprocessing with *D*σ each node has more positive than negative edges, we are guaranteed that *k*_*pn *_≥ *k*/2. In order to compute the expectation value of **s**_σ _on each degree class, we use the self-consistency equation for heterogeneous networks. Following [39], the self-consistency equation on the degree class *k *is given by(4)

where(5)

is the effective "field magnetization" of each node from its neighboring nodes and the subindex σ in **s **indicates that the value is computed in the gauge transformed basis. The use of *k*_*pn *_instead of the degree *k *corrects the equations (4)-(5) for the frustration of the system. In practice, for our gauge transformed networks the number of negative signs is at most 20% (often much less), meaning that *k*_*pn *_~ *k *for most degree classes. From (4) and (5), we have an expression for the mean field expectation value ⟨**s**_σ_⟩ weighted with respect to the degree classes:

and, neglecting fluctuations around each ⟨**s**_σ_⟩ the mean field Hamiltonian is(6)

As , the energy is invariant to the gauge transformation *D*_σ_. In fact, from **s**_σ _= *D*_σ_**s**, we have . Hence the mean field calculations for _σ _are valid also in the original . In addition, however, as **s**_σ,*ground *_= **1**, in the gauge transformed system we have that, as *β *→ ∞, ⟨**s**_σ_⟩ → 1, a property which is in general not verified in the original basis, which will instead concentrate at its own ground state . Therefore, for all practical purposes, ⟨**s**_σ_⟩ can be taken as "order parameter" of the spin glass. In fact, since the gauge transformation minimizes the number of negative edges, it also maximizes the number of spins whose value is +1 in the ground state. Hence, just like in a ferromagnet (i.e., in a spin system in which for all nonzero *J*_*ij *_one has *J*_*ij *_= +1), the average value of **s**_σ _(i.e., the "magnetization") tends to 1 when the system is "cooled".

## Authors' contributions

GI and CA participated in the conceiving of the study, in the statistical analysis and in the drafting of the manuscript. Both authors read and approved the final manuscript.

## Supplementary Material

Additional file 1 **Supplementary Material**. Supplementary notes, figures and tables are provided in this additional pdf file. Click here for file
