# Supplementary material for: Monotonicity, frustration, and ordered response: an analysis of the energy landscape of perturbed large-scale biological networks
Source: BMC Syst Biol. 2010 Jun 10;4:83. doi: 10.1186/1752-0509-4-83 (PMC2909160; doi:10.1186/1752-0509-4-83)
Supplement: Additional file 1 — Supplementary Material. Supplementary notes, figures and tables are provided in this additional pdf file. [file 1752-0509-4-83-S1.PDF]

SUPPLEMENTARY NOTES
for
Monotonicity, frustration, and ordered response: an analysis of the energy landscape of perturbed large-scale biological networks

G. Iacono and C. Altafini*

SISSA Int. School for Advanced Studies
via Beirut 2-4, 34014 Trieste, Italy

February 23, 2010

Contents

1 Representing transcriptional, signaling and metabolic networks as signed graphs 2

2 Description of the algorithms for the computation of the frustration index δ 3

3 Statistical tests 5

4 Analytic computation of internal energy and magnetization: the feedforward loop example 6

List of Figures

S1 . . . 10
S2 . . . 12
S3 . . . 12
S4 . . . 13
S5 . . . 13
S6 . . . 14
S7 . . . 15
S8 . . . 15
S9 . . . 16
S10 . . . 16
S11 . . . 17
S12 . . . 17
S13 . . . 18

*Corresponding author: altafini@sissa.it

1 Representing transcriptional, signaling and metabolic networks as signed graphs

In a transcriptional regulatory network, an edge represents the link between a gene product acting as a transcription factor and a target gene, and lumps together all the intermediate steps required to carry out the synthesis of a new mRNA. For prokaryotes, for example, these include the binding of the transcription factor to the DNA, the recruitment of a polymerase, the unwinding of the DNA helix, the detachment of the σ-factor and the conformational changes in the polymerase preceeding elongation, the release of both the DNA and of the complete mRNA at the termination phase. Taking into account explicitely the kinetic details of all these steps for each transcriptional event is computationally prohibitive, hence the choice of lumping them into a single causal action is *de facto* without alternatives. The role as activator or repressor is captured by the sign of the edge. The activator sign means that in presence of the transcription factor the mRNA synthesis occurs, while it does not when the transcription factor is absent (the opposite for an inhibitor). Energetically, the difference between the two alternatives is considerable, as it implies the occurring or less of all the intermediate biochemical reactions mentioned above.

Various types of representations are possible for a signaling pathway, [12], for example at functional or stoichiometric level of detail. At the functional level, the propagation of a signal is described by means of activatory and inhibitory effects among the molecular components, while at the stoichiometric level, the details of the biochemical reactions leading to these effects are included. Most of the reactions of a signaling network are binding interactions (e.g. protein-protein or ligand-protein) and catalytic reactions (e.g. phosphorylation/dephosphorylation, GEF/GAP, etc.). The functional representation elucidates better the causal transfer of "information" along the pathways, as the details about the chemical transformations may hamper the readability of a pathway chart. The energetic content of this functional representation is however different from that for transcriptional networks, where an activator/inhibitor link decides whether a new macromolecule of mRNA is created/not created out of raw material. Here the causal link represents only the propagation of a signal, and corresponds for example to the transmission of a phosphate group from a donor to a receiver protein. The difference in energy between a phosphorylated and a dephosphorylated version of the same protein roughly corresponds to the energetic cost needed to catalyze the reaction. Hence, for our purposes, it is of interest to consider the details of the reaction kinetics that lead to this cause-effect action, as they give a more precise description, both biochemically and energetically, of the signaling processes. See also [17, 3, 8] for other considerations on the approach followed. Large scale signaling networks providing such a level of description are available for example in [11],[10]. These two are the models used in our work. Other models of this kind appear in [9] and [13] and produce similar results. The stoichiometric level of detail is also the default choice for metabolic networks, where the network itself is usually assembled from basic chemical reactions. A wealth of such networks is available in B. Palsson's group web site, see http://gcrg.ucsd.edu/In_Silico_Organisms. We decided to consider the two metabolic networks of *E.coli* and *S.cerevisiae*. Tests with other metabolic networks give essentially the same results. Reference publications are [14] for *E.coli*, and [5] for *S.cerevisiae*.

At stoichiometric level, the basic building blocks for our signaling and metabolic networks are reactions such as

$$X_1 + \ldots + X_p \rightleftharpoons Z_1 + \ldots + Z_q$$

where X_i and Z_i are chemical species (nodes of our network). While in signaling networks these are essentially all proteins, in metabolic networks the only chemical species considered are metabolites. In order to translate this type of biochemical reactions into a signed graph, we

follow the same approach as [3]. The simplest possible nontrivial such reaction is bimolecular

$$X_1 + X_2 \rightleftharpoons Z_1 \tag{S1}$$

and its associated kinetics is

$$\begin{aligned} \frac{dx_1}{dt} &= -f_1(x_1, x_2) + f_2(z_1) \\ \frac{dx_2}{dt} &= -f_1(x_1, x_2) + f_2(z_1) \\ \frac{dz_1}{dt} &= f_1(x_1, x_2) - f_2(z_1) \end{aligned} \tag{S2}$$

where $f_i(\cdot)$ can have various functional forms, such as mass-action, Michaelis-Menten, or cooperative Hill. Consider the formal Jacobian of (S2)

$$\mathcal{A} = \begin{bmatrix} -\frac{\partial f_1}{\partial x_1} & -\frac{\partial f_1}{\partial x_2} & \frac{\partial f_2}{\partial z_1} \\ -\frac{\partial f_1}{\partial x_1} & -\frac{\partial f_1}{\partial x_2} & \frac{\partial f_2}{\partial z_1} \\ \frac{\partial f_1}{\partial x_1} & \frac{\partial f_1}{\partial x_2} & -\frac{\partial f_2}{\partial z_1} \end{bmatrix}.$$

For the functional forms mentioned above, we have the following signed adjacency matrix

$$\mathrm{sign}(\mathcal{A}) = \begin{bmatrix} -1 & -1 & 1 \\ -1 & -1 & 1 \\ 1 & 1 & -1 \end{bmatrix}$$

which leads, once we disregard the diagonal terms [17], to

$$\mathcal{J} = \begin{bmatrix} 0 & -1 & 1 \\ -1 & 0 & 1 \\ 1 & 1 & 0 \end{bmatrix}$$

whose graph is a single frustrated cycle of 3 nodes and 3 edges. For this dynamical system, every possible spin assignement leaves one inconsistent edge, which is enough to conclude that the system is not monotone. In terms of dynamical behavior, this means that there is no globally ordered way for the system to respond to perturbations. For this particular case, the lack of order can be easily understood from the dynamics of the system (S1)-(S2) with respect to an equilibrium state. When more product Z_1 is added, both the reagents X_1 and X_2 increase owing to the backward dissociation reaction. However, when more of one of the reagents e.g. X_1 is added to the equilibrium state, the concentration of the other reagent X_2 decreases because the excess of X_1 moves the equilibrium of the reaction, hence depleting more X_2. Thus in this simple prototype system the lack of monotonicity is due to the process of compound formation/destruction.

2 Description of the algorithms for the computation of the frustration index δ

This Section briefly summarizes the procedures described in [7] to estimate the true value of the frustration index δ. For a undirected, connected[1] network of n nodes and m edges, the number

[1] Our networks are not connected, but they all have a single very large connected component, see Table S2. The remaining components have few, if any, cycles, and are always trivial for the purposes of computing δ.

of fundamental cycles is $\mu = m - n + 1$, and each cycle of the network can be expressed as a linear combination of the fundamental cycles (i.e., the elements of a basis of the cycle space, see [4]). As each fundamental cycle is univocally characterized by a chord (i.e., an edge in the complement of the spanning tree with respect to which the fundamental cycles are being computed), if the sign of a fundamental cycle is negative, then changing sign to its chord renders the cycle positive. Since all linear combinations of positive fundamental cycles yield positive cycles or edge-disjoint positive cycles, μ is an upper bound on the frustration index δ. Another, unrelated, upper bound for δ is provided by the theory of signed graphs as $(m - \sqrt{m})/2$ [16]. Hence denoting $\delta_{max} = \min\{\mu, (m - \sqrt{m})/2\}$, we have that $\delta \leqslant \delta_{max}$ for any signed network of given n, m. Computing exactly δ is an NP-hard problem, equivalent to the well-known MAX-CUT problem [3], or to the problem of finding the ground state of a frustrated spin system in statistical physics [17]. Various approaches to the problem for biological networks have been proposed, normally formulated as the identification of the minimal number of edges whose removal leave the graph monotone, see e.g. [3] and [6]. Our approach consists in finding, for the signed adjacency matrix $\mathcal{J}$, a (diagonal) signature matrix D_σ, $\sigma = \begin{bmatrix}\sigma_1 & \ldots & \sigma_n\end{bmatrix}$, $\sigma_i \in \{\pm 1\}$, such that $D_\sigma \mathcal{J} D_\sigma$ has the least possible number of negative signs. Any such D_σ is a change of sign through a cut set of the graph of $\mathcal{J}$. Such operations are alternatively called switching equivalences in the theory of signed graphs [19], or gauge transformations in the theory of frustrated spin systems [18], and correspond to changes in the partial order relationship in the theory of monotone systems [17, 15]. A system is monotone if and only if $\exists\ \sigma$ such that $\mathcal{J}_\sigma = D_\sigma \mathcal{J} D_\sigma$ has all entries $\geqslant 0$. When a system is not monotone, computing its distance to monotonicity (i.e., its frustration) is a hard problem, as is well known from the theory of frustrated spin systems. Unlike the usual algorithms to find the ground states in spin systems, which explore the space of spin configurations $\mathbf{s}$, our procedure works on the adjacency matrix $\mathcal{J}$. For example in the yeast cell cycle example of Fig. 1 of the paper, the "gauge transformation" achieving the global minimum of negative signs in $\mathcal{J}_\sigma$ is a sign switch through the cut set isolating Clb1,2 and Cln1,2 and Clb5,6 from the rest of the network. It can be observed that in the original graph the aforementioned 3 nodes have all more negative than positive edges. As searching over all cut sets of the graph is exponentially hard, seeking for "local" cut sets partitioning a graph into 1 and $n-1$ nodes is the basic heuristic we adopt in the algorithms. The big advantage of miminizing the negative entries of $\mathcal{J}_\sigma$ is that no matter if the procedure stops in a local or global minimum, it simplifies the localization of the potential frustrations, as one of the optimal spin assignements (ground states) of $\mathcal{J}_\sigma$ is always $\mathbf{s}_{\sigma,ground} = \mathbf{1}$, i.e., all spins up (and by global symmetry, all spins down, $\mathbf{s}_{\sigma,ground} = -\mathbf{1}$). These residual negative signs of $\mathcal{J}_\sigma$ can for example be associated to the chords of a basis of fundamental cycles (by choosing a spanning tree that avoids them whenever possible), hence each frustration gets univocally associated with a single fundamental cycle. Furthermore, on an adjacency matrix composed largely by positive edges it gets easier to try to associate to each remaining negative edge a cycle completed only by means of positive edges. As long as such frustrated cycles are built edge-disjoint, then the frustration they carry can be shown to be uneliminable. This allows to compute also a lower bound for δ for each $\mathcal{J}_\sigma$. Each run of the algorithm stops in a minimum which can be local or global. Metropolis-like rules allow to explore locally the landscape. After each termination, the algorithm is randomly re-initialized and the whole procedure repeated. Fig. S3 shows the distribution of random initial conditions for the 3 networks mostly discussed in the paper. Overall each spin gets an average very close to 50% of assignements equal to -1. Also plotting the Hamming distance among these random instances confirms that the search space (of dim 2^n) is uniformely (though not exhaustively) explored. On this iterated search, our heuristic algorithms exhibit performances that are both efficient computationally (millions of runs in a few minutes) and effective, where effectiveness is provable by showing that the

uncertainty gap in the candidate values for δ (i.e., the difference between the best upper and lower bounds found) are always much more reduced than for example in the approximation algorithms of [3] where a semidefinite programming approach *à la* Goemans-Williamson is used. Table 2 of the paper shows the values obtained for the 8 networks considered in this study.

The number of iterations of the algorithms on each of the 8 networks is shown in Table S3, where also the number of distinct global minima reached is provided. Two global minima are distinct if they correspond to different signed adjacency matrices $\mathcal{J}_\sigma$. Notice that "trivial" σ different only for sign changes on the leaves of the graph (which for transcriptional networks constitute 40-70% of the nodes) are not included, as they are not taken into account by the algorithm.

For 5 of the 8 networks, the gap between lower and upper bound for δ is nonempty, see Table 2 of the paper. Since our algorithm attain the same value for the candidate minimum (i.e., δ_{up} in Table 2) hundreds or thousands of times, it is much likely that this residual gap is due to the imprecise computation of the lower bound δ_{low}. As a matter of fact, this computation is reminescent of the so-called "cycle packing" problem, which is also known to be NP-hard [2].

3 Statistical tests

Z-statistics for δ with respect to a null model To evaluate how significant the frustration index δ is, we compare this value with the one obtained by means of a null model. The null model is obtained by randomly reshuffling the signs of the edges on the nodes, while maintaining the same number of positive and negative edges of the original graph. A gaussian distribution for the frustration index, of mean δ_{null} and standard deviation σ_{null}, is obtained from 1000 random model instances. For each network, the Z-score of this null model is given by

$$\text{Z-score} = (\delta - \delta_{null})/\sigma_{null}.$$

The parameters of these null models and the p-value of the Z-score for the 8 networks are shown in Fig. 2 and in Table 2 of the paper.

Sign packing The sign packing index ϕ corresponds to the total number of edges belonging to nodes enriched in positive or in negative signs. A p-value is calculated for each of the nodes. The nodes with a p-value less or equal than a given significance threshold θ are classified as enriched and their degree is added to the sign packing index. The p-value is calculated with respect to a null model network obtained by random shuffling the edges. Let v be a node with total degree d and positive degree d^+, then the probability of finding for the node v an amount ℓ of positive edges less or equal to d^+ in the null model counterpart is:

$$p(\ell \leq d^+) = \sum_{i=0}^{d^+} \binom{d}{i} p^i \cdot (1-p)^{d-i} \tag{S3}$$

where p is the probability of the event "positive edge", and it is equal to the ratio between the number m_+ of positive edges and the total number of edges m found in the original network: $p = m_+/m$. A p-value $\to 0$ means that there is a significant amount of negative edges and a significant lack of positive edges. An analogous formula is used for the analysis of negative edge enrichment. Choosing as threshold $\theta = 10^{-70}$, we have the values of ϕ and Z-score of Table S6, where, as above,

$$\text{Z-score} = (\phi - \phi_{null})/\sigma_{null}$$

GO enrichment The GO enrichment for the transcriptional network of *E.coli* was carried out by means of a simple hypergeometric test using the GO annotations updated to 7/8/2009 (42491 annotations). As background for the enrichment, we used the set of all the 126 transcription factors, obtaining the p-values shown in Table S5. Moreover, to further compare double function (D) transcription factors with those that are only activators and repressors (A/R), we changed the background to the set of all the genes and we compared the enrichment of the two groups D and A/R against such background. The terms previously shown to be significant for double function transcription factors still remain significant also when changing background. More in detail, both groups of single function and double function transcription factors were enriched with respect to the background of all the genes. The ratio between the p-values obtained for the A/R group and the p-values obtained for the D group were calculated. The terms previously shown to be significant for double function transcription factors still remain significant also when changing background. Furthermore, many of the terms are not significantly enriched (NE) in the group of activators/repressors suggesting that they are peculiar of double function transcription factors. The remaining terms, enriched in both the groups, always show a much higher p-value for the double function transcription factors set, again confirming the significance of the enrichment carried out.

4 Analytic computation of internal energy and magnetization: the feedforward loop example

In this Section we consider the so-called feedforward loop, i.e., a 3-node motif recurrent in some networks [1]. The example is so simple that it can be completely solved analytically and therefore illustrates well all the concepts introduced and studied in the paper.

Consider the two networks in Fig. S2. The one in (a) is called coherent feedforward loop and has associated signed directed adjacency matrix

$$\mathcal{J}_{\text{ns,coh}} = \begin{bmatrix} 0 & 0 & 0 \\ -1 & 0 & 0 \\ 1 & -1 & 0 \end{bmatrix},$$

while the one in (b) is called incoherent feedforward loop and its matrix J is

$$\mathcal{J}_{\text{ns,incoh}} = \begin{bmatrix} 0 & 0 & 0 \\ -1 & 0 & 0 \\ 1 & 1 & 0 \end{bmatrix}.$$

The coherent feedforward loop is a monotone system. To see it, simply consider the symmetrized $\mathcal{J}_{\text{coh}} = \mathcal{J}_{\text{ns,coh}} + \mathcal{J}_{\text{ns,coh}}^T$ and apply a sign change through the cut set isolating the second node from the rest of the graph (blue cut set in Fig. S2 (a)): $D_\sigma = \text{diag}\{1, -1, 1\}$. This implies that J_σ has all nonnegative entries. On the contrary, no such D_σ exists for the incoherent feedforward loop, meaning that it is not monotone.

For the $2^n = 8$ configurations of the possible values of the s_i, $i = 1, \dots 3$, the distributions of $h(\mathbf{s})$ in the two systems are as follows

$$h_{\text{coh}} = \begin{cases} -3 & 2 \text{ times} \\ 1 & 6 \text{ times} \end{cases}$$

and

$$h_{\text{incoh}} = \begin{cases} -1 & 6 \text{ times} \\ 3 & 2 \text{ times.} \end{cases}$$

As expected, the coherent feedforward loop (a monotone system) has (up to a global symmetry i.e., a sign flip on all spins) a single "ground state" ($\mathbf{s} = [1 \ -1 \ 1]^T$), while the incoherent feedforward loop (a non-monotone systems) has a degenerate "ground state" of multiplicity 3 (6 counting also the global symmetry).

For a given β ("inverse temperature"), the partition function $Z = \sum_{s_i \in \{\pm 1\}} e^{-\beta h(\mathbf{s})}$ is, respectively,

$$Z_{\text{coh}} = 6e^{-\beta} + 2e^{3\beta}$$

and

$$Z_{\text{incoh}} = 2e^{-3\beta} + 6e^{\beta}.$$

Hence, the probabilities of the two ground states

$$p(\mathbf{s}_{\text{ground}}) = \frac{e^{-\beta h(\mathbf{s}_{\text{ground}})}}{Z}$$

are

$$p_{\text{coh}} = \frac{e^{3\beta}}{6e^{-\beta} + 2e^{3\beta}} \quad \text{and} \quad p_{\text{incoh}} = \frac{e^{\beta}}{2e^{-3\beta} + 6e^{\beta}}.$$

These probabilities are drawn as a function of β in Fig. S2 (c). For $\beta \to 0$ ("infinite temperature" limit) all states are equiprobable. Increasing β (i.e., "lowering the temperature") the coherent feedforward loop has a much higher probability of being found in an assigned ground state than the incoherent one (top plot), althought this last will be more often found in any of its more numerous ground states (bottom plot), due to the small size of the system.

While the sum of h_{coh} and h_{incoh} over all 8 possible assignements of $\mathbf{s}$ is 0, the expectation value of h, defined as

$$\langle h \rangle = -\frac{\partial \ln Z(\beta)}{\partial \beta} = -\frac{1}{Z(\beta)} \frac{\partial Z(\beta)}{\partial \beta}$$

for the two feedforward loops corresponds to

$$\langle h_{\text{coh}} \rangle = \frac{-6\left(e^{3\beta} - e^{-\beta}\right)}{6e^{-\beta} + 2e^{3\beta}} \quad \text{and} \quad \langle h_{\text{incoh}} \rangle = \frac{-6\left(e^{\beta} - e^{-3\beta}\right)}{2e^{-3\beta} + 6e^{\beta}}.$$

From the plot of Fig. S2 (d), the coherent feedforward loop always maintains a lower $\langle h \rangle$ for all $\beta > 0$, meaning that indeed perturbations propagate through the network in a coherent manner, i.e., with consistent signs whenever they reach the fan-in node (i.e., on the cycle of the undirected graph).

References

[1] U. Alon. *An Introduction to Systems Biology - Design Principles of Biological Circuits.* Chapman & Hall/CRC;, 2006.

[2] A. Caprara, A. Panconesi, and R. Rizzi. Packing cycles in undirected graphs. *J. Algorithms*, 48(1):239–256, 2003.

[3] B. DasGupta, G. A. Enciso, E. Sontag, and Y. Zhang. Algorithmic and complexity results for decompositions of biological networks into monotone subsystems. *Biosystems*, 90(1):161–178, 2007.

[4] N. Deo. *Graph theory with applications to engineering and computer science.* Prentice-Hall, Englewood Cliffs, N. J., 1974.

[5] J. Förster, I. Famili, P. Fu, B. Ø. Palsson, and J. Nielsen. Genome-scale reconstruction of the *Saccharomyces cerevisiae* metabolic network. *Genome Res.*, 13(2):244–253, 2003.

[6] F. Hüffner, N. Betzler, and R. Niedermeier. Separator-based data reduction for signed graph balancing. *Journal of Combinatorial Optimization*, page (to appear), 2009.

[7] G. Iacono, F. Ramezani, N. Soranzo, and C. Altafini. Determining the distance to monotonicity of a biological network: a graph-theoretical approach. *IET Systems Biology*, page to appear, 2010.

[8] A. Ma'ayan, R. Iyengar, and E. Sontag. Proximity of intracellular regulatory networks to monotone. *IET Systems Biology*, 2:103–112, 2008.

[9] K. Oda, T. Kimura, Y. Matsuoka, A. Funahashi, M. Muramatsu, and H. Kitano. Molecular interaction map of a macrophage. *AfCS reports*, 2(14), 2004.

[10] K. Oda and H. Kitano. A comprehensive map of the toll-like receptor signaling network. *Mol. Syst. Biol.*, 2:2006.0015, 2006.

[11] K. Oda, Y. Matsuoka, A. Funahashi, and H. Kitano. A comprehensive pathway map of epidermal growth factor receptor signaling. *Mol Syst Biol*, 1:2005, 2005.

[12] J. A. Papin, T. Hunter, B. O. Palsson, and S. Subramaniam. Reconstruction of cellular signalling networks and analysis of their properties. *Nat Rev Mol Cell Biol*, 6(2):99–111, Feb 2005.

[13] J. A. Papin and B. O. Palsson. The JAK-STAT signaling network in the human B-cell: an extreme signaling pathway analysis. *Biophys J*, 87(1):37–46, 2004.

[14] J. L. Reed, T. D. Vo, C. H. Schilling, and B. Ø. Palsson. An expanded genome-scale model of *Escherichia coli* K-12 (*i*JR904 GSM/GPR). *Genome Biol.*, 4(9):R54, 2003.

[15] H. L. Smith. Systems of ordinary differential equations which generate an order preserving flow. A survey of results. *SIAM Review*, 30(1):87–113, 1988.

[16] P. Solé and T. Zaslavsky. A coding approach to signed graphs. *SIAM J. Discrete Math.*, 7(4):544–553, 1994.

[17] E. D. Sontag. Monotone and near-monotone biochemical networks. *Systems and Synthetic Biology*, 1:59–87, 2007.

[18] G. Toulouse. Theory of the frustration effect in spin glasses : I. *Communications on Physics*, 2:115, 1977.

[19] T. Zaslavsky. Signed graphs. *Discrete Appl. Math.*, 4(1):47–74, 1982.

Table S1: Networks used in this study and their original signed edges. n and m are the number of nodes and edges of the oriented graph. m_{symm} is the number of edges in the resulting nonoriented graph.

Network	n	m	incompat./symm. edges pairs	pos./neg. edges	m_{symm}
transcriptional					
E.coli	1475	3320	4/5	1881/1339	3215
Yeast	690	1082	1/0	860/220	1080
B.subtilis	918	1324	2/2	985/339	1318
Cory	344	366	0/0	72/294	366
signaling					
EGRF	330	852	4/65	515/264	779
Toll-like	679	2204	1/413	1020/769	1789
metabolic					
E.coli	757	6116	108/1949	2809/1142	3951
Yeast	797	4436	23/1494	2084/812	2896

Table S2: Basic data for the eight biological networks. n_{cc} and m_{cc} are the number of nodes and the number of edges of the largest connected component of a network. The positive and negative fundamental cycles refers to $\mathcal{J}_\sigma$. The leaves are the nodes not involved in any cycle.

Network	conn. comp.	n_{cc}/m_{cc}	fund. cyc.	+ fund. cyc.	− fund. cyc.	leaves
transcriptional						
E.coli	21	1376/3150	1775	1404	371	556
Yeast	11	664/1064	401	360	41	348
B.subtilis	15	886/300	415	344	71	528
Cory	26	182/224	48	39	9	264
signaling						
EGRF	2	313/755	452	259	193	12
Toll-like	2	672/1780	1112	644	468	59
metabolic						
Yeast	9	780/2887	2108	1361	747	84
E.coli	1	757/3951	3195	2178	1017	17

Table S3: Number of runs and number of distint global minima found by the algorithms. In the last column the minima are expressed as a percentage over the iterations.

Network	Iterations	Global minima	fraction minima/iterations
transcriptional			
E.coli	3000000	23126	0,77%
Yeast	1000000	2292	0,23%
B.Subtilis	1000000	25793	2,58%
Cory	500000	33940	6,78%
signaling			
EGFR	3000000	8018	0,26%
Toll-like	1000000	298	0,03%
metabolic			
Yeast	3000000	11471	0,37%
E.coli	1000000	30591	3,05%

Table S4: Order thresholds for the eight biological networks. The value of β for which $\langle \mathbf{s}_\sigma \rangle > 0.8$ is taken in the paper as a characteristic threshold for the concentration of occupancy probability to the ground state. Notice how the values are systematically higher for transcriptional than for signaling/metabolic networks. The ratios are similar for other values of the threshold, e.g., $\langle \mathbf{s}_\sigma \rangle > 0.9$.

Network	$\beta(\langle \mathbf{s}_\sigma \rangle > 0.8)$	$\beta(\langle \mathbf{s}_\sigma \rangle > 0.9)$
transcriptional		
E.coli	0.71	1.02
Yeast	0.81	1.16
B.subtilis	0.90	1.24
Cory	1.05	1.39
signaling		
EGRF	0.46	0.66
Toll-like	0.47	0.69
metabolic		
Yeast	0.45	0.69
E.coli	0.29	0.40

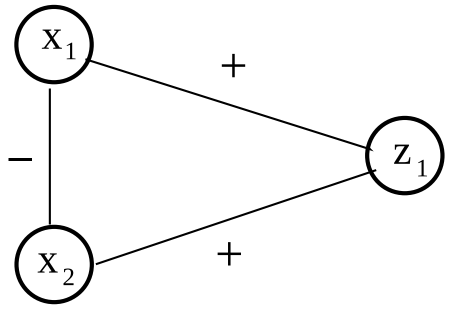

Figure S1: Signed graph for the ODE of (S2).

Table S5: GO categories for the dual role (D) transcription factors for the *E.coli* transcriptional network. The p-value column refers to the p-values obtained comparing dual role transcription factors with the set of all transcription factors as background. The log(p-val(A/R)/p-val(D)) column shows the $\log_{10}$ ratio between the p-values for the two groups A/R and D of transcription factors, see Section 3 for details. NE means not enriched in the A/R group.

GO term	description	p-value	$\log \frac{\text{p-val(A/R)}}{\text{p-val(D)}}$	genes
GO:0000156 (GO:0004871, GO:0060089)	Alters the level of transcription of target genes, usually by binding to a transcription factor, when phosphorylated by a sensor that detects the presence of a particular signal substance outside the cell	$7,37 \cdot 10^{-3}$	12	dpiA, ompR, csgD, rcsA, rcsB, torR, arcA, cpxR, narL, narP, phoB, phoP, rstA
GO:0007165	The cascade of processes by which a signal interacts with a receptor, causing a change in the level or activity of a second messenger or other downstream target, and ultimately effecting a change in the functioning of the cell.	$4,1 \cdot 10^{-3}$	N.E.	dpiA, ompR, tyrR, csgD, sdiA, rcsA, rcsB, torR, arcA, cpxR, narL, narP, phoB, phoP, rstA
GO:0042128	The uptake, from the environment, of nitrates, inorganic or organic salts and esters of nitric acid and the subsequent reduction of nitrate ion to other, less highly oxidized, inorganic nitrogenous substances.	$4,4 \cdot 10^{-2}$	N.E.	nac, narL, narP
GO:0006310	Any process by which a new genotype is formed by reassortment of genes resulting in gene combinations different from those that were present in the parents.In bacteria it may occur by genetic transformation, conjugation, transduction, or F-duction.	$4,5 \cdot 10^{-2}$	N.E.	argR, ihfA, ihfB

Table S6: Sign packing statistics: ϕ is the sign packing index of the original network, ϕ_{null} and σ_{null} the parameters for the gaussian fitting based on a null model with reshuffled edges. In correspondence of a threshold $\theta = 10^{-70}$, the Z-statistics compare ϕ and the null model.

Network	ϕ	ϕ_{null}	σ_{null}	Z_{score}	P-value
transcriptional					
E.coli	1109	627,78	26,56	18,11	$p = 1,14 \cdot 10^{-73}$
Yeast	843	442	18,59	21,56	$p = 1,71 \cdot 10^{-101}$
B.Subtilis	961	438,94	18,8	27,76	$p = 5,13 \cdot 10^{-170}$
Cory	226	100,94	11,54	10,83	$p = 1,15 \cdot 10^{-27}$
signaling					
EGFR	208	219	21,14	-0,52	$p = 0,7$
Toll-like	403	305	23,13	4,2	$p = 1,13 \cdot 10^{-5}$
metabolic					
Yeast	524	581,14	30,35	-1,88	$p = 0,97$
E.coli	405	499,6	37,66	-2,51	$p = 0,994$

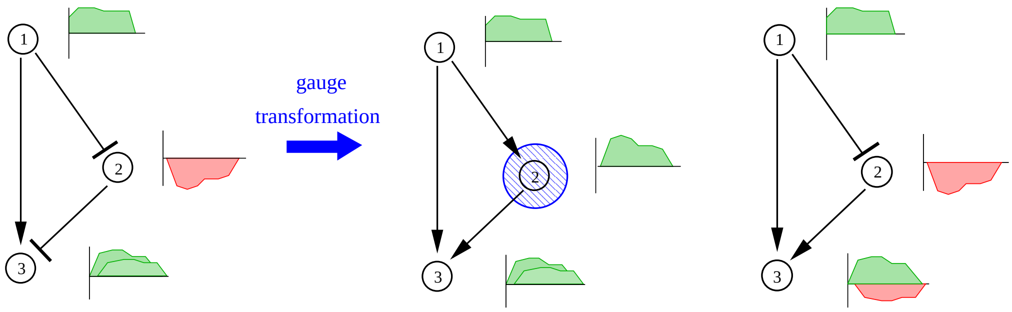

(a) Coherent feedforward. (b) Incoherent feedforward.

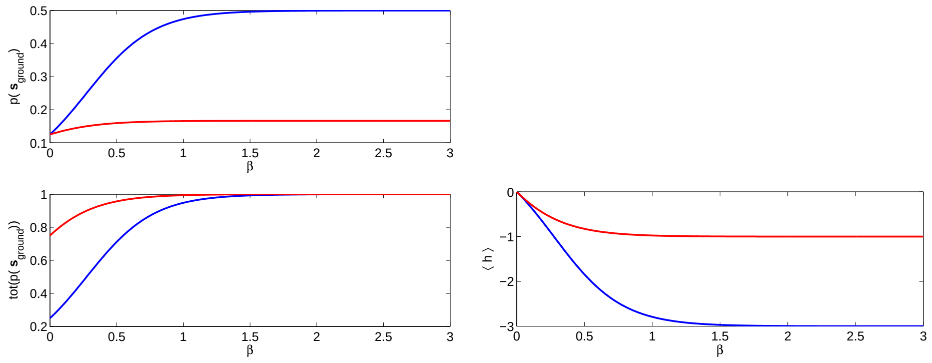

(c) ground state probability (d) average internal energy

Figure S2: Propagation of perturbations along the coherent and incoherent feedforward loops. That the coherent feedforward loop is monotone is seen by applying a gauge transformation (i.e., a sign change) through the node 2: $D_\sigma = \mathrm{diag}\{1, -1, 1\}$. $\mathcal{J}_\sigma = D_\sigma \mathcal{J} D_\sigma$ has all nonnegative entries. The consequence is that while in the first case the perturbations reach the fan-in node with a coherent sign, in the second the signs disagree, meaning that the behavior is less predictable and potentially contradictory. (c) Top: probability of a ground state for both the coherent (blue) and incoherent (red) feedbforward loops as a function of β. Bottom: global probability of a ground state counting multiplicity and also global symmetry (i.e. multiplicity 2 for the coherent and multiplicity 6 for the incoherent), as a function of β. (d) Expectation value of the "energy" h for both the coherent (blue) and incoherent (red) feedforward loops as a function of β.

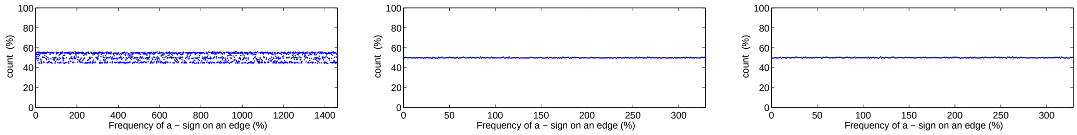

(a) *E.coli-transcr* (b) *EGFR-signal* (c) *Yeast-metab*

Figure S3: Testing uniformity of the distribution in the initial conditions of the gauge transformation algorithms. Shown is the frequency of negative signs on each node of the three networks.

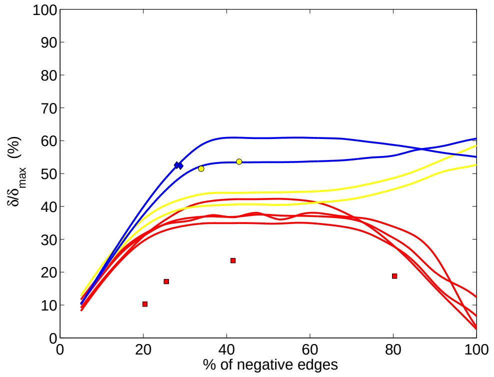

Figure S4: Value of the ratio δ/δ_{max} with varying number of negative edges randomly assigned to each of the 8 networks (mean over 1000 random instances for each of 20 different percentages of negative edges between 5% and 100%). All plots begin growing linearly for low concentration of negative signs, then reach a plateau. For the transcriptional networks (in red) the graph is essentially symmetric around 50% of negative edges. For signaling and transcriptional networks, instead, the second half of the plot is fairly different, with δ remaining high or even increasing. This difference is presumably due to the cycles of length 3 introduced in the construction of these networks. When all signs are negative, all of them are necessarily frustrated. The true values for all networks are also shown (red squares for transcriptional, yellow disks for signaling and blue diamonds for metabolic) and confirm that transcriptional networks are more monotone than expected.

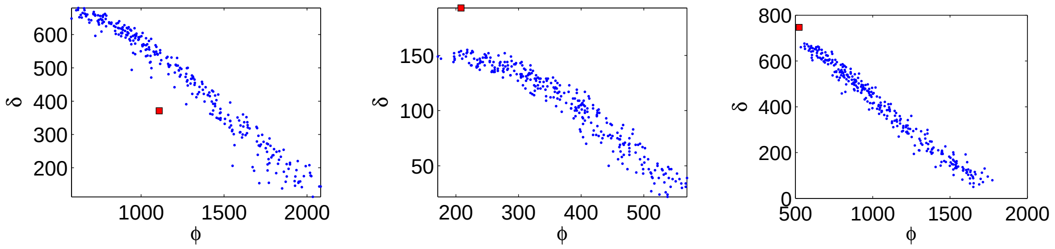

(a) *E.coli-transcr* (b) *EGFR-signal* (c) *Yeast-metab*

Figure S5: Sign packing index versus frustration index on 300 random sign assignements to the edge of 3 of the networks. Each random instance has the same number of negative edges as the true network. Clearly, the higher the sign packing index, the lower the frustration index turns out to be (i.e., the more monotone the system is). Coherently with Fig. 3 of the paper, the true *E.coli-transcr* is an outlier of the distribution (less frustrated than expected from the value of ϕ). Also the *EGFR-signal* is an outlier, but for the opposite reason (δ higher than expected given ϕ).

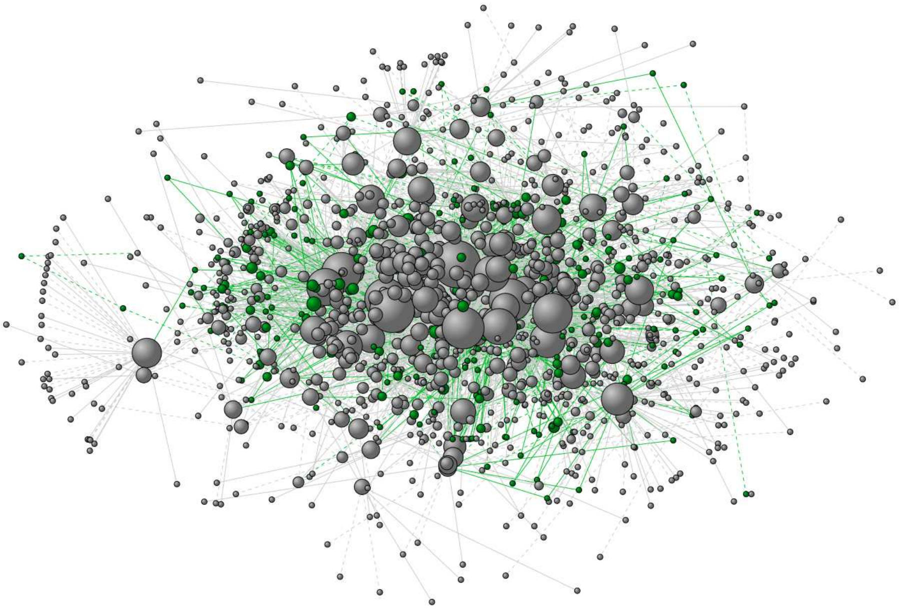

(a) *E.coli-transcr* with randomized signs

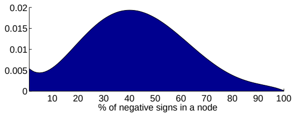

(b) Distribution of randomized signs in *E.coli-transcr*

Figure S6: *E.coli-transcr* network with randomized signs. The sign packing distribution is now gaussian. Compare with Fig. 3 of the paper.

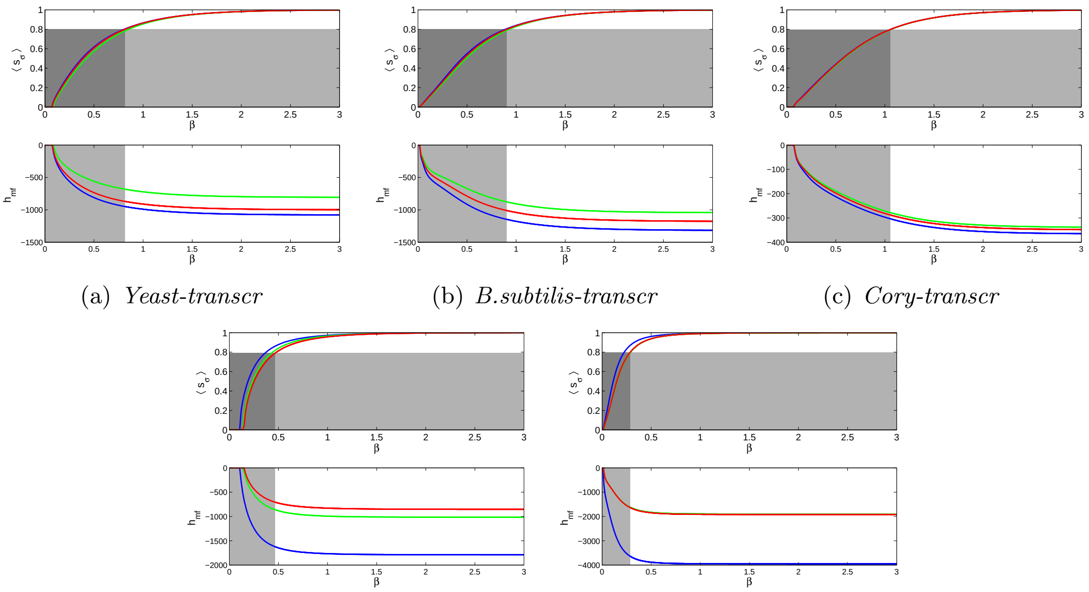

(a) *Yeast-transcr* (b) *B.subtilis-transcr* (c) *Cory-transcr*

(d) *Toll-like-signal* (e) *E.coli-metab*

Figure S7: Mean field magnetization $\langle \mathbf{s}_\sigma \rangle$ (in the gauge transformed basis) and energy h_{mf} for the 5 networks not shown in Fig. 4 of the paper, as a function of β. The behavior observed is in complete agreement with Fig. 4. The transcriptional networks achieve order for larger values of β than the signaling/metabolic networks, and this property is essentially independent of the level of frustration.

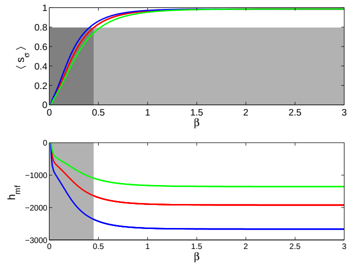

Figure S8: Mean field magnetization $\langle \mathbf{s}_\sigma \rangle$ (in the gauge transformed basis) and energy h_{mf} for the transcriptional network of *E.coli* without nodes that are leaves. Compare with Fig. 4(a) of the paper.

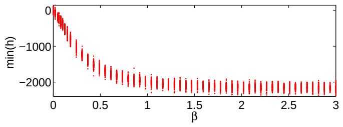

(a) *E.coli-transcr*

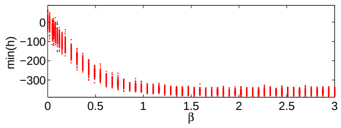

(b) *EGFR-signal*

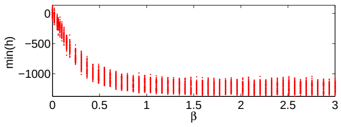

(c) *Yeast-metab*

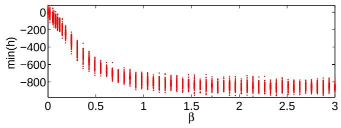

(d) *Yeast-transcr*

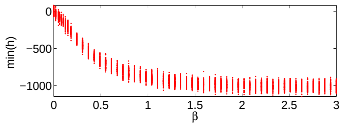

(e) *B.subtilis-transcr*

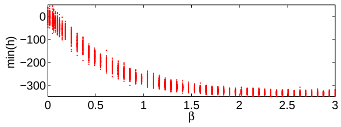

(f) *Cory-transcr*

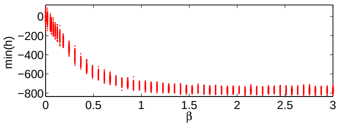

(g) *Toll-like-signal*

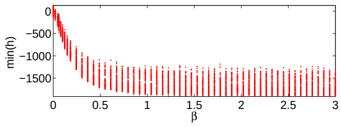

(h) *E.coli-metab*

Figure S9: Metropolis-Montecarlo simulations for the 8 networks. For each value of β, shown are the endpoints of 100 trajectories, each composed of 10000 random moves (each move is a spin flip). A move decreasing energy is always accepted; a move increasing energy is accepted with a probability $e^{-\beta \Delta h}$, where $\Delta h > 0$ is the energetic increment. As β increases, the moves of positive energy are more penalized, and the trajectories "concentrate" at the low energies. Notice that a certain difference in the β-thresholds for order between transcriptional and signaling/metabolic networks is still visible, although less than in the mean field calculations. In part this is due to the fact that the absolute minimum found with Metropolis trajectories is usually not a global minimum. For example for *E.coli-transcr* the global minimum in correspondence of our $\delta = 371$ is $h(\mathbf{s}) = -2473$, while the minimum of the Metropolis trajectories is $h(\mathbf{s}) = -2375$ i.e., $\delta_{Metropolis} = 449$, or 5% of the spins (78) incorrectly flipped in the best case.

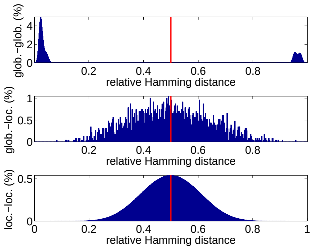

(a) *Yeast-transcr*

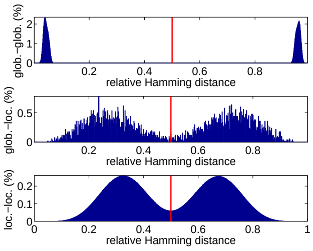

(b) *B.subtilis-transcr*

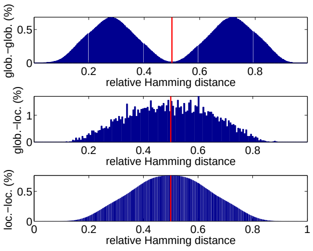

(c) *Cory-transcr*

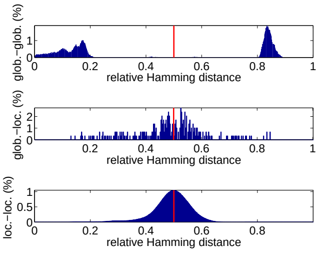

(d) *Toll-like-signal*

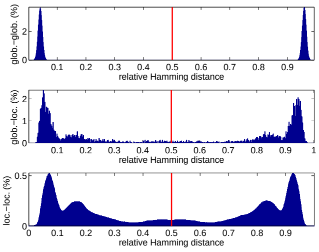

(e) *E.coli-metab*

Figure S10: Relative Hamming distance between pairs of minima for the 5 networks not shown in Fig. 5.

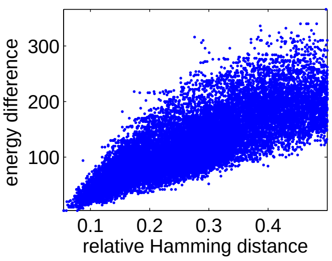

(a) *E.coli-transcr*

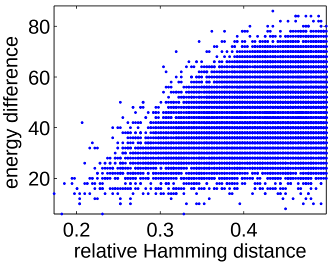

(b) *EGFR-signal*

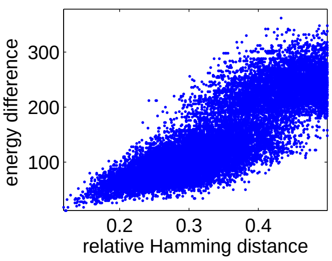

(c) *Yeast-metab*

Figure S11: Difference in energy between local and global minima as a function of the relative Hamming distance. The lower envelope of this energetic difference is linearly correlated with distance for *E.coli* but not for *EGFR* and *Yeast-metab*, where the wells of global minima are surrounded by local minima of comparable energy.

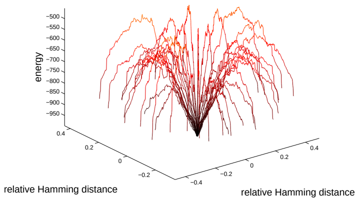

(a) *Yeast-transcr*

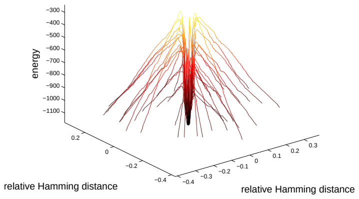

(b) *B.subtilis-transcr*

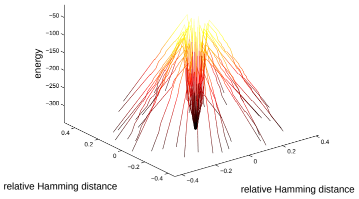

(c) *Cory-transcr*

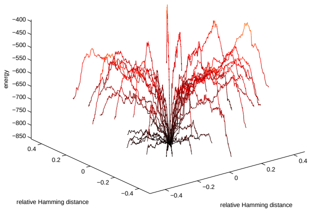

(d) *Toll-like-signal*

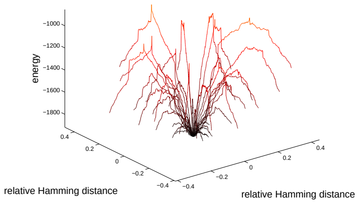

(e) *E.coli-metab*

Figure S12: Montecarlo trajectories connecting a global minimum to its surrounding local minima for the 5 networks not shown in Fig. 6. The trajectories are represented as emanating from a single point and radially distributed. As in Fig. 6, in the transcriptional networks the global minima are surrounded by high barriers, no matter whether they are localized in a thin well (*Yeast* and *B.subtilis*) or not (*Corynebacterium*). On the contrary, both *Toll-like* and *E.coli-metab* show numerous low-energy paths connecting global and local minima.

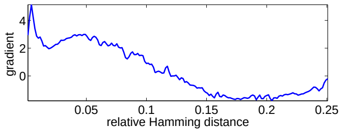

(a) *Yeast-transcr*

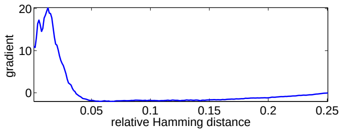

(b) *B.subtilis-transcr*

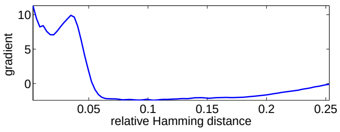

(c) *Cory-transcr*

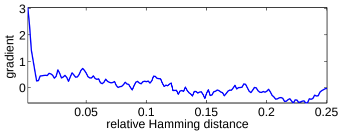

(d) *Toll-like-signal*

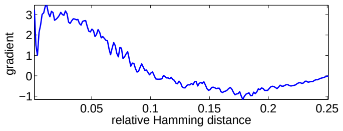

(e) *E.coli-metab*

Figure S13: Average gradient over 1000 Montecarlo trajectories connecting a global and a local minimum for the 5 networks of Fig. S12. Notice how for the transcriptional networks the steep and funneled landscape around the global optima is confirmed (especially the unusual ones for *B.subtilis* and *Corynebacterium*).
